# Wearing a surgical mask during chemotherapy session is safe

**DOI:** 10.1038/s41598-025-94940-z

**Published:** 2025-03-27

**Authors:** Ali Alkan, Mert Şahin, Ece Dilan Bozkurt, Aslı Alkan, Özgür Tanrıverdi

**Affiliations:** 1 Department of Medical Oncology, Muğla Sıtkı Koçman University School of Medicine, Kötekli Mah. Marmaris Yolu Bulvarı No: 55 Menteşe, 48000 Muğla, Türkiye; 2 Department of Internal Medicine, Muğla Sıtkı Koçman University School of Medicine, Muğla, Türkiye; 3 Department of Anesthesiology and Reanimation, Division of Intensive Care Medicine, Muğla Sıtkı Koçman University School of Medicine, Muğla, Türkiye

**Keywords:** Surgical mask, Blood gases, Cancer, Chemotherapy, Carbon dioxide, Oncology, Cancer, Cancer therapy

## Abstract

Surgical masks(SM) have become essential to our daily lives with the COVID-19 pandemic. It is recommended as the cheapest, most effective preventive method. The effects of SM on patients receiving chemotherapy are unknown. Our study aimed to investigate the effects of SM on oxygenation and CO2 retention in cancer patients receiving chemotherapy and to examine its possible clinical consequences. Patients diagnosed with cancer and receiving chemotherapy were included in the study. Venous blood gas, SO2 by pulse oximeter, and vital signs were recorded before and after treatment. Acute toxicities encountered during treatment were recorded. One hundred twenty-six patients with a median age of 60 (33–85) were evaluated in the study. The comparison of pre-post treatment parameters showed statistically significant changes in Ph (7.37 vs. 7.35, *p* < 0.01), pCO2 (44.2 vs. 45.8, *p* = 0.049), HCO3 (25.7 vs. 25.3, *p* = 0.003), SpO2 (97.0 vs. 96.0, *p* = 0.08), fever (36.4 vs. 36.3, *p* = 0.023). All the changes were clinically insignificant and in normal ranges. Chemotherapy-related acute toxicity was noted in 4 (3.2%) of the patients. Lung morbidity, cancer type, lung metastasis status, treatment applied, duration of therapy, and acute toxicity do not affect the current parameters. In our study, it was shown that constantly wearing a SM in patients receiving chemotherapy caused CO2 retention and a tendency to hypoxemia. However, the current changes were clinically insignificant and within the normal range. Surgical masks can be used safely in cancer patients receiving systemic treatment.

## Introduction

Following the emergence of the COVID-19 infection in China in 2020, the use of surgical masks (SM) in daily life has become widespread based on the recommendations of the World Health Organization and health authorities to prevent the dissemination of the virus.Current scientific studies support usingSM to reduce viral transmission^1^.Based on numerous data, the use of SMs to prevent the spread of COVID-19 has become widespread. SMshave been recommended as the cheapest and most effective protective method during the pandemic.However, potential adverse effects associated with SM use have become a research subject.There are studies in the literature, particularly concerning potential hypoxemia and hypercapnia that may arise due to ventilation problems.Some studies have shown that SMs usage does not cause oxygenation issues or CO2 retention.The Impact of SMs usage on the development of CO2 retention has also been investigated using various methods.One study involved 50 healthy volunteers wearing surgical and reusable regular face masks during 10-minute sitting and walking periods, followed by saturation and CO2 levelmeasurements using transdermal devices^2–4^.The results showed no significant differences in SPO2 and CO2 retention among participants.Contrary to some studies, reports indicate that mask ventilation may cause hypoxemia, associated with fatigue.Furthermore, many studies have shown that it could lead to hypercapnia^5,6^.

When considering the importance of COVID-19 for cancer patients, these patients form the most vulnerable population due to tumor burden and the immunosuppressive effects of anticancer agents.Indeed, there is scientific evidence supporting this.For example, in a cohort study of 105 cancer patients, lung cancer was reported to be the most common cancer histology in infected patients, followed by gastrointestinal cancer (12.38%) and breast cancer (10.48%)^7^.Another study involving 28 COVID-19 cancer patients found that individuals with stage 4 disease had a higher frequency of infection^8^.The study by Liang et al. reported a higher incidence of severe clinical conditions (ICU admission, intubation requirement, death) associated with COVID-19 among cancer patients compared to the general population^9^.In light of this information and data, using SMs is paramount for protecting cancer patients from COVID-19 and also from other air-borne infections.The use of masks is essential in chemotherapy units where cancer patients have to come into contact with both high-risk healthcare workers and cancer patients who have to receive chemotherapy in terms of COVID-19 and need to visit regularly^10^.However, multiple mask-related side effects can be seen in cancer patients using masks.Literature data examining the side effects of SM usage in the general population are limited^11^.In the previous four years, to protect themselves from the pandemic, cancer patients, especially within hospitals, have continuously used SM.Patients receiving continuous treatment in chemotherapy units for durations ranging from 30 min to 6 h are closely monitored for treatment-related acute toxicity. To avoid air-borne infections, SM are actively used during chemotherapy sessions. Given that even in the general population, continuous use of SM can lead to numerous side effects, similar adverse effects are inevitable during chemotherapy.Additionally, potential hypoxemia or hypercapnia and their adverse clinical outcomes are also unavoidable.There is no literature study investigating this topic.

Our study aimed to investigate the effects of SM on oxygenation and CO2 retention in cancer patients undergoing chemotherapy and to examine the potential impact of SM on acute toxicity during chemotherapy.

### Methods

Our study is prospective and was approved by the Clinical Research Ethics Committee of Muğla Sıtkı Koçman University on 24.08.2022 with decision number 14/VIII.The study was conducted in accordance with the Declaration of Helsinki. The patients who are more than 18 years of age, with a histopathologically documented cancer diagnosis and receiving regular chemotherapy every 1, 2, 3, or 4 weeks and every three months, were included.The patients whose planned chemotherapy could not be completed, who removed their mask during treatment for reasons other than toxicity, were on continuous oxygen therapy and needed to wear a mask other than a surgical mask due to comorbidities, were excluded from the study. In addition, any patient who had supplemental oxygen during session, was planned to be dropped out of the study.

Patients included in the study were invited to participate by the researchers after routine pre-chemotherapy evaluations. The patients provided consent to participate the study and signed the informed consent form, were included. Signed informed consent was obtained from the participants. Patients who agreed were assessed according to the “patient evaluation form,” and relevant information regarding their disease and treatments was recorded by reviewing their medical records.The patient evaluation form included patient characteristics, accompanying comorbidities, medications used, disease characteristics, treatment details, and previously experienced acute or chronic toxicities.Venous blood gas samples and oxygen saturation (SpO2) measurements were taken from patients who met the inclusion criteria and agreed to participate in the study.Patients were monitored for compliance with the research and any complications that developed during chemotherapy while wearing SM.After the completion of chemotherapy, SpO2 measurements, venous blood gas sampling and vital parameters were repeated.Blood gas samples were analyzed using the ABL 90 Flex blood gas analyzer.SpO2 measurements were conducted using the EDAN H100B Pulse oximeter.The data obtained from these measurements were recorded in the patient evaluation form.

The statistical analyses of the study were performed using SPSS (Statistical Package for Social Sciences) for Windows 22 (Armonk, NY: IBM Corp.) software package.Before detailed statistical analyses, the data distribution was examined using the Kolmogorov-Smirnov test and skewness-kurtosis coefficients.Non-parametric tests were used to study non-normally distributed groups, while parametric tests were used for normally distributed groups.Descriptive statistics included number (%), median, mean, standard deviation, and highest, and lowest values.Independent Sample T-tests and Mann-Whitney U tests were used to compare two independent groups. Pearson’s chi-square or Fisher’s exact test was used to compare *categorical data*.Patients’ clinical characteristics were categorized during the analysis to test their effects on descriptive statistics and endpoints.Patients were classified as elderly and young based on an age threshold of 65 years.Patients with at least one comorbidity were categorized as “having comorbidity.” To evaluate the Impact of the number of medications, patients actively using five or more medications were considered to have “polypharmacy.” To assess the effects of lung morbidity related to the primary disease, patients were categorized as “with/without metastasis,” lung metastases as “unilateral/bilateral,” and the number of metastases as “high/low” with a threshold of five.The duration of follow-up under treatment was categorized as “long/short treatment” based on a median duration of 150 (60–410) minutes.

## Results

Between 01.11.22 and 30.06.23, 126 cases who applied to our center for chemotherapy were included in the study.The characteristics of the patients werepresented in Table 1.The median age was 60 (33–85) years and 56.3% were female.93 patients (73.8%) had at least one comorbidity.The most common primary cancer was breast cancer (41, 32.5%).Lung metastasis was present in 35 (27.8%) patients, with 12 (34.3%) of these having bilateral metastasis.Half of the patients receiving active treatment (63, 50.0%) were on palliative chemotherapy. The most common regimen was a three-week regimen (71, 56.3%), with patients frequently receiving platinum-based (50, 39.7%) and taxane-based (42, 55.3%) treatments.The median follow-up duration was 150 min (60–410).

**Table 1:** Patient, disease and treatment characteristics.

Characteristics	N(%)
Gender	
Female	71 (56.3)
Male	55 (43.7)
Age	
Mean	59.9 +/- 10.9
Median (range)	60 (33-85)
Old (≥65)	47 (37.3)
Young (<65)	79 (62.7)
Comorbidity present	93 (73.8)
Hypertension	57 (45.2)
Diabetes Mellitus	20 (23.0)
Coronary heart disease	26 (20.6)
Thyroid Disease	10 (7.9)
COPD/ Asthma	21 (16.7)
Other	31 (24.6)
Polypharmacy (≥5 drugs)	104 (82.5)
Inhaler treatment- present	19 (15.1)
Diagnosis	
Breast Cancer	41 (32.5)
Lung Cancer	29 (23.0)
Colorectal Cancer	11 (8.7)
Upper GIS Cancer	13 (10.3)
Gynecological Cancer	15 (11.9)
Other	17 (12.5)
Lung metastasis- present	35 (27.8)
Lung metastasis location	
Unilateral	23 (65.7)
Bilateral	12 (34.3)
Number of Lung metastasis	
<5	27(77.1)
≥5	8 (22.9)
Therapy	
Neoadjuvant	28 (22.2)
Adjuvant	35 (27.8)
Palliative	63 (50.0)
Treatment frequency (every)	
1 week	15 (11.9)
2 weeks	35(27.8)
3 weeks	71 (56.3)
4 weeks	5 (4.0)
Treatment modality	
Platine based	50 (39.7)
Taxane-based	42 (33.3)
Anti-Her-2	21 (16.7)
Fluoropyrimidines	19 (15.1)
Immunotherapy	10 (7.9)
Anthracycline- based	6(4.8)
Treatment duration (min)	
Mean	172.5 +/- 76.9
Median(range)	150 (60- 410)
Treatment duration category	
Long (>150 min)	62 (49.2)
Short (<150min)	64 (50.8)

COPD: chronic obstructive pulmonary disease, GIS: Gastrointestinal system.

The physical examination findings of patients before chemotherapy were normal.No hemodynamic instability or signs of infection were detected in any of the patients receiving chemotherapy.The median pH before and after chemotherapy was 7.37 vs. 7.35, with a median difference of -0.02 (-0.28-0.15),*p* < 0.01 (Table 2).The median CO2 change before and after treatment was 44.25 mmHg vs. 45.8 mmHg, with a median difference of + 1.65 (-30.0-14.6), *p* = 0.019.The median oxygen change before and after treatment was 41.35 mmHg vs. 40.0 mmHg, with a median difference of -1.5 (-1.0-125.0), *p* = 0.15.The median SpO2 change before and after treatment was 97.0 vs. 96.0, with a median difference of 0.00 (-0.9-11.0), *p* = 0.008.The median HCO3 change before and after treatment was 25.75 meq/L vs. 25.30 meq/L, with a median difference of -0.70 (-12.6-11.6), *p* = 0.03.The median lactate change before and after treatment was 1.30 mmol/L vs. 1.20 mmol/L, with a median difference of 0.0 (-2.40-2.5), *p* = 0.46.

**Table 2:** Comparison of blood gas values and vital changes before and after chemotherapy.

Parameters	Median(Range)	Difference (median, range)	p
Ph			
Before	7.37 (7.18- 7.64)	-0.02(-0.28-0.15)	<0.01
After	7.35 (7.26- 7.46)		
pCO2 (mmHg)			
Before	44.25 (22.20- 87.70)	+1.65 (-30.0- 14.6)	0.019
After	45.80 (24.3- 61.00)		
pO2 (mmHg)			
Before	41.35 (14.0- 173.0)	-1.5 (-104.0- 125.0)	0.15
After	40.0 (10.0-150.0)		
SpO2 (%)			
Before	97.0 (85.0- 99.0)	0.00(-9.0- 11.0)	0.008
After	96.0 (84.0- 99.0)		
HCO3 (meq/L)			
Before	25.75 (16.90- 34.1)	-0.70(-12.6- 11.6)	0.03
After	25.30 (14.0- 38.0)		
Lactate (mmol/L)			
Before	1.30 (0.10- 5.90)	0.00 (-2.40- 2.50)	0.46
After	1.20 (0.20- 4.40)		
Pulse (/min)			
Before	81.5 (48- 128)	0.0(-27- 45)	0.20
After	80.0 (46- 125)		
Fever (^o^C)			
Before	36.4 (35.3- 37.2)	0.00(-1.2- 0.50)	0.023
After	36.3 (35.1- 37.0)		
Systolic Blood Pressure (mmHg)			
Before	120.0 (80.0-190.0)	0.0(-40- 35)	0.08
After	120.0 (80.0- 173.0)		
Diastolic Blood Pressure (mmHg)			
Before	70.0 (40.0- 110.0)	0.00 (-40- 30.0)	0.17
After	75.0 (40.0- 110.0)		
Mean Arterial Pressure (mmHg)			
Before	86.70 (53.3- 130.0)	0.0 (-33- 26.7)	0.17
After	90.0 (53.3- 130.0)		

pCO2: partial pressure of carbon dioxide, pO2: partial pressure of oxygen, SpO2: oxygen saturation.

During chemotherapy, four patients developed allergic reactions.However, all these reactions were grade 1 urticaria and they didn’t need O_2_and skin lesions resolved with antihistaminics.The median heart rate change during chemotherapy was 81.5/min before treatment vs. 80.0/min after treatment, with a median difference of 0.0 (-27-45), *p* = 0.20 (Table 2).The median temperature change was 36.4 °C before treatment vs. 36.3 °C after treatment, with a median difference of 0.0 (-1.2-0.50), *p* = 0.023.The median systolic blood pressure change was 120.0 mmHg before treatment vs. 120.0 mmHg after treatment, with a median difference of 0.0 (-40-35), *p* = 0.08.The median diastolic blood pressure change was 70.0 mmHg before treatment vs. 75.0 mmHg after treatment, with a median difference of 0.0 (-40-30), *p* = 0.17.The median mean arterial pressure change was 86.7 mmHg before treatment vs. 90.0 mmHg after treatment, with a median difference of 0.0 (-33-26.7), *p* = 0.17.

The parameters affecting changes in blood gas and vital signs during the treatment process were evaluated.The effects of clinical features, disease characteristics, and treatment features were analyzed by comparing median changes.In the analysis of parameters affecting pH change, a more significant pH decrease was observed in females compared to males (-0.02 vs. -0.002, *p* = 0.03) (Table 3).In our study, analyzing the factors affecting CO2 increase, it was found that the CO2 increase was higher in females (2.4 vs. 0.2 mmHg, *p* = 0.087), although this did not reach statistical significance.In patients receiving anti-Her2 treatment, the CO2 increase was found to be higher compared to those not receiving this treatment (4.6 vs. 1.0 mmHg, *p* = 0.005).Correspondingly, HCO3 showed an increase in those receiving anti-Her2 treatment (0.6 vs. -1.0 meq/L, *p* < 0.001).All patients receiving anti-Her2 treatment were female (21 patients, 29.6% of the females).The median age of these patients was 53 years (33–75), with a median treatment duration of 140 min (90–341).Only 2 (9.5%) of the patients were aged 65 and above, and 10 (47.6%) had at least one comorbidity, but none had bronchial asthma or COPD.All of these patients had breast cancer and frequently received treatment every three weeks (20, 95.2%).None of the patients experienced acute toxicity during treatment.The median CO2 level before treatment was 43.7 mmHg (34.1–57.8) and 47.7 mmHg (37.8–61.0) after treatment (*p* = 0.01).

**Table 3:** The effect of Clinical, pathological and treatment characteristics on parameters.

	Ph	pCo2(mmHg)	pO2(mmHg)	HCO3(meq/L)	Lactate(mmol/L)					
Parameters	Median(Range)	p	Median(Range)	p	Median(Range)	p	Median(Range)	p	Median(Range)	p
Gender										
Female	-0.02(-0.2-0.07)	0.03	2.4(-13.2-14)	0.087	-7(-84-125)	0.014	-0,4(-12,6-11.6)	0.09	-0,1(-1.9-2.5)	0.81
Male	-0.002(-0.1-0.15)		0.2(-30- 14.6)		5(-104-108.3)		-1.1(-9.3-9.6)		0.0(-2,4-1,4)	
Age										
Young (<65)	-0.02(-0.09-0.13)	0.33	1.7(-23.5-14)	0.65	-3(-50-125)	0.70	-0.04(-9.3-11.6)	0.072	0(-2.4-1.8)	0.81
Old ( >65)	-0.03(-0.28-0.15)		1.4(-30-14)		0(-104-103.3)		-1.2(-12.6-9.6)		0(-1.9-2.5)	
Comorbidity										
Absent	-0.02(-0.09-0.07)	0.92	1.7(-13.2-12.30)	0.66	-3.2(-48-125)	0.72	-0.5(-4.7-11.6)	0.20	0(-1.7-1.8)	0.67
Present	-0.02(-0.28-0.15)		1.6(-30-14.6)		-0.9(-104-108)		-0.8(-12.6-9.6)		0(-2.4-2.5)	
COPD/ Asthma										
Absent	-0.02(-0.16-0.15)	0.64	1.3(-30-14.6)	0.37	-1(-65-125)	0.82	-0.7(-9.3-11.6)	0.63	0(-1.7-2.5)	0.61
Present	-0.01(-0.28-0.12)		3.8(-11.2-12.5)		-4.1(-104-20.8)		-1(-12.6-9.6)		0(-2.4-1.5)	
Polypharmacy										
Absent	-0.0215(-0.08-0.04)	0.53	2.9(-11.2-8.5)	0.39	-6.5(-48-34)	0.58	-0.25(-3.5-11.6)	0.41	0(-1.1-1.8)	0.73
Present	-0.02(-0.28-0.15)		1.5(-30-14.6)		-0.95(-104-125)		-0.75(-12.6-9.6)		0(-.2.4-2.5)	
İnhaler treatment										
Absent	-0.02(-0.16-0.13)	0.39	1.8(-23.5-14.6)	0.76	-3(-65-125)	0.13	-0.7(-9.3-11.6)	0.48	0(-1.7-2.5)	0.84
Present	-0.01(-0.28-0.15)		1.6(-30-12.5)		7.7(-104-108)		-1.5(-12.6-9.6)		0(-2.4-1.3)	
Diagnosis										
Breast	-0.03(-0.06-0.0)		2.3(-1.9-6.5)		-16(-35-3)		0.35(-0.7-1.4)		-0.4(-0.5- -0.3)	
Lung	0.007(-0./9-0.15)	0.12	-0.4(-30-7.2)	0.082	11(-104-108.3)	0.084	-1.55(-.3.2-9.6)	0.23	0.15(-1.7-1.3)	0.60
Colorectal	-0.01(-0.05-0.02)		-.0.6(-8.5-5.4)		13(-65-34)		-.1.5(-.3.5-0.6)		0.2(-0.5-1.8)	
Upper GIS	-0.002(-0.08-0.02)		3.6(-5-6.9)		-14(-30-2)		0(-1.4-0.2)		-0.5(-1.1-0)	
Gynecological	-0.04(-0.08-0.0)		1.25(1.2-1.3)		-10.5(-14-7)		-0.9(-2.6-0.8)		0.85(0.6-1.1)	
Other	-0.05(-0.16-0.05)		7.1(-11.2-14.6)		-10.5(-36-11)		1.55(-3-2.3)		0.05(-0.5-0.9)	
Lung	0.007(-0.09-0.15)	0.43	-0.4(-30-7.2)	0.91	11(-104-108)	0.81	-1.55(-3.2-9.6)	0.89	0.15(-1.7-1.3)	0.82
Other	-0.03(-0.16-0.06)		2.4(-14.2-14.6)		-7(-65-34)		0(-3.5-2.3)		-0.2(-1.1-1.8)	
Therapy										
Neoadjuvant	-0.215(-0.08-0.05)	0.68	1.65(-16.5-8.1)	0.77	-3.1(-50-99)	0.94	-1.1(-9.3-2.4)	0.26	0.1(-2.4-1.8)	0.22
Adjuvant	-0.03(-0.09-0.04)		1(-11.2-12.5)		-4(-62-39)		-.0.7(-3.8-4.9)		0.2(-1.9-1.8)	
Palliative	-0.011(-0.28-0.15)		2.3(-30-14.6)		0(-104-125)		-0.4(-12.6-11.6)		-0.1(-1.7-2.5)	
Lung metastasis										
Present	-0.01(-0.16-0.15)	0.71	1.9(-30-14.6)	0.87	3(-104-108.3)	0.35	-0.7(-3.5-9.6)	0.82	0(-1.7-1.8)	0.69
Absent	-0.02(-0.28-0.13)		1.6(-23.5-14)		-3.2(-84-125)		-0.7(-12.6-11.6)		0(-2.4-2.5)	
Number of Lung metastasis										
<5	-0.002(-0.16-0.15)	0.77	2,4(-30-14.6)	0.80	2(-104-108.3)	0.47	-0.6(-3.5-9.6)	0.19	0(-1.7-1.8)	0.19
≥5	-0.035(-0.07-0.03)		0.5(-6.5-8.4)		10(-36-31)		-1.75(-3.4-1.9)		0.35(-0.5-1.3)	
Treatment frequency (every)										
1 week	-0.03(-0.08-0.04)	0.72	1,416.7-4.5)	0.40	2(-30-53)	0.54	-1.1(-7.3-3.4)	0.40	-0.1(-1.9-1.5)	0.92
2 weeks	-0.02(-0.16-0.12)		0.2(-16.5-14.6)		-2(-104-108.3)		-0.6(-9.3-9.6)		0(-1.1-1.8)	
3 weeks	-0.02(-0.1-0.15)		2.4(-30-14)		-5(-62-125)		-0.5(-7.6-11.6)		0(-2.4-2.5)	
4 weeks	0(-0.28-0.03)		-1.9(-6.5-4.1)		3(-84-14)		-1.5(-12.6-1.5)		-0.3(-0.5-1)	
Treatment modality										
Platine										
Absent	-0.02(-0.28-0.15)	0.92	1.95(-30-14.6)	0.77	-0.5(-104-125)	0.66	-0.45(-12.6-11.6)	0.075	-0.1(-1.9-2.5)	0.44
Present	-0.0115(-0.09-0.07)		1.6(-16.5-14)		-2(-65-99)		-1(-9.3-4.9)		0.2(-2.4-1.5)	
Taxane										
Absent	-0.02(-0.28-0.15)	0.96	1.3(-30-14.6)	0.64	-2(-104-108.3)	0.77	-0.55(-12.6-11.6)	0.41	-0.15(-2.4-1.8)	0.017
Present	-0.0155(-0.1-0.06)		1.75(-16.7-14)		-0.55(-62-125)		-1(-7.3-4.9)		0.2(-1.4-2.5)	
Anti-Her-2										
Absent	-0.011(-0.28-0.15)	0.13	-1(-30-14.6)	0.005	0(-104-125)	0.076	-1(-12.6-9.6)	<0.001		0.51
Present	-0.03(-0.09-0.02)		4.6(-7-12.3)		-11(-48-39)		0.6(-1.8-11.6)		0(-2.4-2.5)0(-1.7-1.6)	
Fluoropyrimidine										
Absent	-0.02(-0.28-0.15)	0.43	1.8(-30-14.6)	0.36	-3(-104-125)	0.045	-0.7(-12.6-11.6)	0.52	0(-2.4-2.5)	0.27
Present	-0.002(-0.06-0.05)		-0.6(-16.5-8)		7(-65-99)		-0.8(-9.3-1.9)		0.2(-1.1-1.8)	
Immunotherapy										
Absent	-0.02(-0.28-0.15)	0.25	1.65(-30-14.6)	0.98	-1.5(-84-125)	0.94	-0.7(-12.6-	0.25	0(-2.4-2.5)	0.27
Present	-0.01(-0.06-0.12)		1.65(-12.6-7.2)		-2.5(-104-108.3)		11.6)-0.35(-2.5-9.6)		-0.25(-1.1-1)	
Anthracycline										
Absent	-0.02(-0.28-0.15)	0.41	1.6(-30-14.6)	0.28	-0.95(-104-125)	0.67	-0.75(-12.6-11.6)	0.23	0(-2.4-2.5)	0.69
Present	-0.025(-0.06- -0.01)		3.55(-0.1-8.5)		-5.5(-22-8)		0.3(-1.2-1.2)		-0.2(-0.5-0.6)	
Treatment duration category										
Long (>150 min)	-0.01(-0.1-0.12)	0.34	0.3(-16.7-14)	0.10	-3.6(-104-125)	0.85	-0.95(-9.3-9.6)	0.099	0.2(-1.1-2.5)	0.003
Short (<150min)	-0.025(-0.28-0.15)		2.9(-30-14.6)		0(-84-89)		-0.3(-12.6-11.6)		-0.2(-2.4-1.8)	

COPD: chronic obstructive pulmonary disease, pCO2: partial pressure of carbon dioxide, pO2: partial pressure of oxygen, SpO2: oxygen saturation, GIS: Gastrointestinal system.

**Table 4:** The Effect of Clinical, Pathological and Treatment Characteristics on SpO2 and vital parameters.

	Ph	pCo2(mmHg)	pO2(mmHg)	HCO3(meq/L)	Lactate(mmol/L)					
Parameters	Median(Range)	p	Median(Range)	p	Median(Range)	p	Median(Range)	p	Median(Range)	p
Gender										
Female	-0.02(-0.2-0.07)	0.03	2.4(-13.2-14)	0.087	-7(-84-125)	0.014	-0,4(-12,6-11.6)	0.09	-0,1(-1.9-2.5)	0.81
Male	-0.002(-0.1-0.15)		0.2(-30- 14.6)		5(-104-108.3)		-1.1(-9.3-9.6)		0.0(-2,4-1,4)	
Age										
Young (<65)	-0.02(-0.09-0.13)	0.33	1.7(-23.5-14)	0.65	-3(-50-125)	0.70	-0.04(-9.3-11.6)	0.072	0(-2.4-1.8)	0.81
Old ( >65)	-0.03(-0.28-0.15)		1.4(-30-14)		0(-104-103.3)		-1.2(-12.6-9.6)		0(-1.9-2.5)	
Comorbidity										
Absent	-0.02(-0.09-0.07)	0.92	1.7(-13.2-12.30)	0.66	-3.2(-48-125)	0.72	-0.5(-4.7-11.6)	0.20	0(-1.7-1.8)	0.67
Present	-0.02(-0.28-0.15)		1.6(-30-14.6)		-0.9(-104-108)		-0.8(-12.6-9.6)		0(-2.4-2.5)	
COPD/ Asthma										
Absent	-0.02(-0.16-0.15)	0.64	1.3(-30-14.6)	0.37	-1(-65-125)	0.82	-0.7(-9.3-11.6)	0.63	0(-1.7-2.5)	0.61
Present	-0.01(-0.28-0.12)		3.8(-11.2-12.5)		-4.1(-104-20.8)		-1(-12.6-9.6)		0(-2.4-1.5)	
Polypharmacy										
Absent	-0.0215(-0.08-0.04)	0.53	2.9(-11.2-8.5)	0.39	-6.5(-48-34)	0.58	-0.25(-3.5-11.6)	0.41	0(-1.1-1.8)	0.73
Present	-0.02(-0.28-0.15)		1.5(-30-14.6)		-0.95(-104-125)		-0.75(-12.6-9.6)		0(-.2.4-2.5)	
İnhaler treatment										
Absent	-0.02(-0.16-0.13)	0.39	1.8(-23.5-14.6)	0.76	-3(-65-125)	0.13	-0.7(-9.3-11.6)	0.48	0(-1.7-2.5)	0.84
Present	-0.01(-0.28-0.15)		1.6(-30-12.5)		7.7(-104-108)		-1.5(-12.6-9.6)		0(-2.4-1.3)	
Diagnosis										
Breast	-0.03(-0.06-0.0)		2.3(-1.9-6.5)		-16(-35-3)		0.35(-0.7-1.4)		-0.4(-0.5- -0.3)	
Lung	0.007(-0./9-0.15)	0.12	-0.4(-30-7.2)	0.082	11(-104-108.3)	0.084	-1.55(-.3.2-9.6)	0.23	0.15(-1.7-1.3)	0.60
Colorectal	-0.01(-0.05-0.02)		-.0.6(-8.5-5.4)		13(-65-34)		-.1.5(-.3.5-0.6)		0.2(-0.5-1.8)	
Upper GIS	-0.002(-0.08-0.02)		3.6(-5-6.9)		-14(-30-2)		0(-1.4-0.2)		-0.5(-1.1-0)	
Gynecological	-0.04(-0.08-0.0)		1.25(1.2-1.3)		-10.5(-14-7)		-0.9(-2.6-0.8)		0.85(0.6-1.1)	
Other	-0.05(-0.16-0.05)		7.1(-11.2-14.6)		-10.5(-36-11)		1.55(-3-2.3)		0.05(-0.5-0.9)	
Lung	0.007(-0.09-0.15)	0.43	-0.4(-30-7.2)	0.91	11(-104-108)	0.81	-1.55(-3.2-9.6)	0.89	0.15(-1.7-1.3)	0.82
Other	-0.03(-0.16-0.06)		2.4(-14.2-14.6)		-7(-65-34)		0(-3.5-2.3)		-0.2(-1.1-1.8)	
Therapy										
Neoadjuvant	-0.215(-0.08-0.05)	0.68	1.65(-16.5-8.1)	0.77	-3.1(-50-99)	0.94	-1.1(-9.3-2.4)	0.26	0.1(-2.4-1.8)	0.22
Adjuvant	-0.03(-0.09-0.04)		1(-11.2-12.5)		-4(-62-39)		-.0.7(-3.8-4.9)		0.2(-1.9-1.8)	
Palliative	-0.011(-0.28-0.15)		2.3(-30-14.6)		0(-104-125)		-0.4(-12.6-11.6)		-0.1(-1.7-2.5)	
Lung metastasis										
Present	-0.01(-0.16-0.15)	0.71	1.9(-30-14.6)	0.87	3(-104-108.3)	0.35	-0.7(-3.5-9.6)	0.82	0(-1.7-1.8)	0.69
Absent	-0.02(-0.28-0.13)		1.6(-23.5-14)		-3.2(-84-125)		-0.7(-12.6-11.6)		0(-2.4-2.5)	
Number of Lung metastasis										
<5	-0.002(-0.16-0.15)	0.77	2,4(-30-14.6)	0.80	2(-104-108.3)	0.47	-0.6(-3.5-9.6)	0.19	0(-1.7-1.8)	0.19
≥5	-0.035(-0.07-0.03)		0.5(-6.5-8.4)		10(-36-31)		-1.75(-3.4-1.9)		0.35(-0.5-1.3)	
Treatment frequency (every)										
1 week	-0.03(-0.08-0.04)	0.72	1,416.7-4.5)	0.40	2(-30-53)	0.54	-1.1(-7.3-3.4)	0.40	-0.1(-1.9-1.5)	0.92
2 weeks	-0.02(-0.16-0.12)		0.2(-16.5-14.6)		-2(-104-108.3)		-0.6(-9.3-9.6)		0(-1.1-1.8)	
3 weeks	-0.02(-0.1-0.15)		2.4(-30-14)		-5(-62-125)		-0.5(-7.6-11.6)		0(-2.4-2.5)	
4 weeks	0(-0.28-0.03)		-1.9(-6.5-4.1)		3(-84-14)		-1.5(-12.6-1.5)		-0.3(-0.5-1)	
Treatment modality										
Platine										
Absent	-0.02(-0.28-0.15)	0.92	1.95(-30-14.6)	0.77	-0.5(-104-125)	0.66	-0.45(-12.6-11.6)	0.075	-0.1(-1.9-2.5)	0.44
Present	-0.0115(-0.09-0.07)		1.6(-16.5-14)		-2(-65-99)		-1(-9.3-4.9)		0.2(-2.4-1.5)	
Taxane										
Absent	-0.02(-0.28-0.15)	0.96	1.3(-30-14.6)	0.64	-2(-104-108.3)	0.77	-0.55(-12.6-11.6)	0.41	-0.15(-2.4-1.8)	0.017
Present	-0.0155(-0.1-0.06)		1.75(-16.7-14)		-0.55(-62-125)		-1(-7.3-4.9)		0.2(-1.4-2.5)	
Anti-Her-2										
Absent	-0.011(-0.28-0.15)	0.13	-1(-30-14.6)	0.005	0(-104-125)	0.076	-1(-12.6-9.6)	<0.001		0.51
Present	-0.03(-0.09-0.02)		4.6(-7-12.3)		-11(-48-39)		0.6(-1.8-11.6)		0(-2.4-2.5)0(-1.7-1.6)	
Fluoropyrimidine										
Absent	-0.02(-0.28-0.15)	0.43	1.8(-30-14.6)	0.36	-3(-104-125)	0.045	-0.7(-12.6-11.6)	0.52	0(-2.4-2.5)	0.27
Present	-0.002(-0.06-0.05)		-0.6(-16.5-8)		7(-65-99)		-0.8(-9.3-1.9)		0.2(-1.1-1.8)	
Immunotherapy										
Absent	-0.02(-0.28-0.15)	0.25	1.65(-30-14.6)	0.98	-1.5(-84-125)	0.94	-0.7(-12.6-	0.25	0(-2.4-2.5)	0.27
Present	-0.01(-0.06-0.12)		1.65(-12.6-7.2)		-2.5(-104-108.3)		11.6)-0.35(-2.5-9.6)		-0.25(-1.1-1)	
Anthracycline										
Absent	-0.02(-0.28-0.15)	0.41	1.6(-30-14.6)	0.28	-0.95(-104-125)	0.67	-0.75(-12.6-11.6)	0.23	0(-2.4-2.5)	0.69
Present	-0.025(-0.06- -0.01)		3.55(-0.1-8.5)		-5.5(-22-8)		0.3(-1.2-1.2)		-0.2(-0.5-0.6)	
Treatment duration category										
Long (>150 min)	-0.01(-0.1-0.12)	0.34	0.3(-16.7-14)	0.10	-3.6(-104-125)	0.85	-0.95(-9.3-9.6)	0.099	0.2(-1.1-2.5)	0.003
Short (<150min)	-0.025(-0.28-0.15)		2.9(-30-14.6)		0(-84-89)		-0.3(-12.6-11.6)		-0.2(-2.4-1.8)	

COPD: Chronic obstructive Pulmonary Disease, DBP: Diastolic blood pressure, MAP: Mean arterial pressure, pCO2: partial pressure of carbon dioxide, pO2: partial pressure of oxygen, SpO2: oxygen saturation, GIS: gastrointestinal system, SBP: Systolic blood pressure,

In the analysis of parameters affecting lactate change, a positive change was observed in patients receiving taxane-based treatment compared to those not (0.2 vs. -0.15 mmol/L, *p* = 0.017).Additionally, a statistically significant positive change was found in patients receiving long-duration treatment (> 150 min) (+ 0.2 vs. -0.2 mmol/L, *p* = 0.003).

In our study, to evaluate mask-related hypoxemia, the analysis of SO2 measurements showed a significant increase in those using inhaler therapy compared to those not using it (+ 1 vs. 0, *p* = 0.032) (Table 4).No statistically significant difference was found in the evaluation of SO2 changes over time between those receiving long and short treatments (*p* = 0.60).No clinically significant changes were observed in vital parameters during treatment.Furthermore, no clinically significant findings were observed in the analysis of the effects of clinical, pathological, and treatment characteristics on vital parameters.

## Discussion

In this study, we found that SM could cause an increase in carbon dioxide levels in cancer patients receiving chemotherapy, based on venous blood gas samples obtained from 126 patients actively undergoing chemotherapy in our hospital’s chemotherapy unit.However, this increase was within reference values and did not cause clinical changes in the patients.

Surgical masks were first used during surgical procedures in 1987^12^.Initially utilized to prevent pathogens from entering the surgical field during exhalation, healthcare workers now actively use masks.Additionally, masks are actively used to reduce the likelihood of airborne pathogen transmission^13^.In December 2019, a new coronavirus causing coronavirus disease 19 (COVID-19) was identified.Subsequently, it spread worldwide, leading to a pandemic.The World Health Organization (WHO) declared the pandemic on March 11, 2020, prompting global efforts to combat it^14^. Although 80% of SARS-CoV-2 infections are asymptomatic, human-to-human transmission occurs via droplets and close contact^15^.In comparison, the patients can present with various clinical presentations, ranging from pneumonia requiring intensive care to sepsis.To date, over 650 million cases and 6.6 million deaths have been reported during the pandemic.Clinically, the virus primarily causes active infections in the upper and lower respiratory tract, but it has also been reported to affect many other systems, causing early and late complications.Although 98.8% of cases recover following an upper respiratory infection, certain groups are at risk.Elderly patients and those with chronic diseases such as cancer, diabetes mellitus, cardiovascular diseases, and chronic lung diseases are at risk for disease complications^16^.Furthermore, COVID-19 infection tends to be more severe in cancer patients than in the general population, with hospitalizations being more frequent and the course of the infection being more fatal.The course of COVID-19 infection in cancer patients can vary based on the type of treatments received, stage, performance score, and the presence of other underlying diseases.This includes patients with cancer and those with hematological malignancies^17–20^.

During the pandemic, before vaccination, hand washing, social isolation, and mask use were the essential tools used to prevent virus dissemination and contamination^13,21^.In April 2020, the WHO issued guidelines recommending SM to prevent the spread of COVID-19^22^.Additionally, studies and reviews in the literature suggest that mask use can reduce the spread of COVID-19 and other respiratory pathogens^23–25^. Surgical mask use has also raised several concerns.These include communication problems, discomfort from prolonged mask use, the need to remove the mask for eating and drinking, and undesirable changes in blood levels of carbon dioxide and oxygen.In our study, we investigated whether mask use during chemotherapy causes hypoxia or hypercapnia in patients actively receiving chemotherapy.

Different types of masks with varying features are recommended in other situations to prevent airborne diseases.However, various problems or complications associated with intermittent or prolonged mask use have been described.Perhaps the most fundamental issue arises from the nature of masks, which provide benefits by creating a mechanical barrier and thus covering the nose and mouth^26^. Potential problems or complications associated with mask use can be summarized as follows^27^: Psychosocial effects, Communication problems, Increased moisture and temperature, Pressure on the nose and ears, Increased respiratory resistance and effort, Hypoxemia, and/or hypercapnia.

The potential side effects of oxygenation and CO2 retention are more concerning with prolonged SM use in both healthy individuals and those with chronic illnesses.Several studies have tested the safety of mask use in patients with chronic lung diseases.In a study by Kyung et al., 97 patients diagnosed with COPD were evaluated for respiratory parameters while wearing N95 masks during 10 min of rest and a 6-minute walk.The study found seven patients could not tolerate the test, and those with FEV1 < 30% or a Medical Research Council dyspnea score of 3 or higher might experience detrimental effects from mask use^28^. In a study by Ciocan et al. involving ten patients, three asthma patients and healthy individuals were tested to determine how mask use affected blood gas parameters during the study.The evaluation showed that there was no significant respiratory impairment in either group.A mask-wearing 4-hour survey did not result in respiratory parameter issues^29^. In a study by Samannan et al., 15 doctors without lung disease and 15 patients with known COPD wore SM during rest and a 6-minute walk test, undergoing pulmonary function and blood gas evaluations^4^.No differences in PCO2 or So2 measurements were found at rest.As expected, a decrease in oxygenation was observed in the COPD group during the 6-minute walk test, but no difference was found in pCO2 values.The study reported that masks could be safely used even in patients with chronic lung diseases. A meta-analysis by Chen et al. showed that while COPD patients wearing masks at rest had a higher respiratory rate, there were no differences in CO2, SO2, or heart rate parameters^30^. In addition to studies emphasizing the safe use of masks in patients with COPD, it has been reported that regular mask use during the pandemic also reduced COPD exacerbations, with a 73.4% decrease in emergency admissions^31^.

No studies in the literature evaluate the possible complications of SM in cancer patients.Even before the pandemic, patients who continuously wore masks became more attached to masks, a vital protection modality during the pandemic.Due to the increased social isolation problem, patients who spend most of their time in the chemotherapy unit while receiving systemic treatment are obliged to wear their masks for extended periods without interruption.However, no studies in the literature evaluate the complications of masks, specifically in cancer patients.In our research, based on the evaluation of venous blood gases and vital parameters before and after treatment, we found a decrease in pH (7.35 vs. 7.37, *p* < 0.001), an increase in CO2 (45.8 vs. 44.2, *p* = 0.019), a decrease in SO2 (96 vs. 97, *p* = 0.008), and a decrease in HCO3 (25.3 vs. 25.7, *p* = 0.03).These statistically significant changes highlight the potential numerical effects of SM in patients receiving chemotherapy.However, these changes are subclinical, without clinical significance, and did not require additional intervention during treatment in our cohort of 126 patients.Although pCO2 values in our cohort remained within the normal range, the median pCO2 was 44.2 mmHg before treatment.These values might be close to the upper limit due to the patients’ continuous mask use even before treatment.Patients often wear their masks intermittently or continuously while waiting for their turn, being evaluated in the clinic, and starting treatment.This could explain the high baseline pCO2 values.Since our study aimed to assess mask-related effects and complications during chemotherapy, we did not intervene in patients’ mask use before treatment.Additionally, patients did not spend any mask-free period before starting treatment to avoid altering the routine practice effects.

In our study, subgroup analyses were performed, assuming that patients with primary lung cancer and those with underlying chronic lung diseases might be affected by prolonged mask use.Potential SM effects were tested by categorizing patients as lung cancer/other, with/without lung metastasis, with metastasis number > 5/<5, and with/without COPD/asthma.The analysis found no adverse differences in these groups’ oxygenation, CO2 retention, or vital parameters.In evaluating the effects of different parameters, no statistical difference was found in geriatric or palliative care patients.Subgroup analysis revealed that female patients experienced statistically insignificant more CO2 increases (2.4 vs. 0.2 mmHg, *p* = 0.087). There is no specific study about the impact of gender on CO2 retention, but it can be attributed to basic physiology and differences of total lung capacities between females and males. In addition, CO2 increase was higher in patients receiving anti-Her2 treatment than those not (4.6 vs. 1.0 mmHg, *p* = 0.005).This group, all women with breast cancer, showed no distinguishing characteristics discussed in detail in the results section.No literature evidence was found to explain the higher CO2 retention in this predominantly trastuzumab-treated cohort.Anti-Her2 monoclonal antibodies are the backbone treatment modalities of Her2 expressing breast cancer^32^. Interstitial pneumonitis is a rare toxicity of trastuzumab and its drug conjugates^33^. However, in the DESTINY-Breast02 study, the most common treatment-emergent adverse events associated with drug discontinuation were pneumonitis (6%) and interstitial lung disease(4%)^34^. Apart from subacute and chronic pulmonary side effects of anti-Her2 therapies, there is no specifically defined acute transfusion related hypercapnia in the literature. HER2 is expressed by type II pneumocytes and is involved in cell proliferation and wound repair. So, by anti-Her2 therapies, repair mechanisms and surfactant secretion in the lungs, may be disrupted. These hypothetical mechanisms can be the causes of our findings. Hovewer, this current situation warrants further investigation in future studies.

### Limitations

In our scientific study, venous blood gas sampling was chosen instead of arterial blood gas sampling due to concerns that patients would experience more pain during the arterial blood gas sampling process, as arteries are more profound than veins, and the procedure takes longer, potentially affecting patient compliance.Although there are differences between the values measured in arterial and venous blood gases, these differences are more pronounced in partial oxygen pressure^35^.We attempted to compensate for this difference in partial oxygen pressure by measuring oxygen saturation with a pulse oximeter before and after the procedure.Indeed, studies are showing and recommending that saturation sampling alone, when performed simultaneously with venous blood gas sampling, can replace arterial blood gas sampling.

Many types of masks are available on the market, with the use of normal cloth masks and SM being more common in the community than other types.In our study, we chose to use SM.Further studies could investigate how different types of masks affect blood gas and SpO2 parameters in chemotherapy patients.Our study primarily focused on patients receiving chemotherapy for lung, breast, upper gastrointestinal, and colorectal cancers, with insufficient sample sizes to evaluate results in patients with other types of cancer.Patients were monitored in different rooms during their chemotherapy sessions, and the ventilation conditions of each room were not the same, with varying distances from the entrance.Standardizing this technically is challenging, but theoretically, changes related to ventilation could have affected the results to some extent.Most of the treatment regimens included steroids as premedication, but the doses could vary, and steroids were not present, especially in patients receiving immunotherapy.The effects of steroids on the airway might have had a minimal impact on the results. A design with a control group could have provided us better results about the impact of SM. But, at the time of the study because of the anxiety of COVID pandemic and fragility of our patients, all our patients preferred to wear surgical masks. In the following years, it can be tested with a control arm.

## Conclusion

Surgical mask use is an effective and inexpensive method for preventing diseases transmitted via aerosols.However, literature suggests intermittent or prolonged use of masks can cause mechanical and psychosocial problems, as well as issues with oxygenation and CO2 retention.There is a lack of data on this subject for cancer patients who frequently use masks in their daily practice.Our study examined the effects of SM worn by patients undergoing chemotherapy on blood gas and vital parameters.It was found that continuous use of SM in patients receiving chemotherapy caused a decrease in pH, an increase in CO2, a decrease in SO2, and a decrease in HCO3.However, these changes were clinically insignificant and occurred within normal ranges.Subgroup analysis revealed no additional risk in patients with lung comorbidities, primary lung cancer, or lung metastases.Surgical masks can be safely used by cancer patients receiving systemic treatment.

## Data Availability

The datasets used and analyzed during the current study are available from the corresponding author on reasonable request.
